# Antiretroviral Drug Activity in Macaques Infected during Pre-Exposure Prophylaxis Has a Transient Effect on Cell-Associated SHIV DNA Reservoirs

**DOI:** 10.1371/journal.pone.0164821

**Published:** 2016-11-02

**Authors:** Mian-er Cong, Chou-Pong Pau, Walid Heneine, J. Gerardo García-Lerma

**Affiliations:** Laboratory Branch, Division of HIV/AIDS Prevention, National Center for HIV/AIDS, Viral Hepatitis, STD, and TB Prevention, Centers for Disease Control and Prevention, Atlanta, Georgia, United States of America; CEA, FRANCE

## Abstract

**Background:**

Pre-exposure prophylaxis (PrEP) with emtricitabine and tenofovir disoproxil fumarate (FTC/TDF) is a novel HIV prevention strategy. Suboptimal PrEP adherence and HIV infection creates an opportunity for continued antiretroviral drug activity during undiagnosed infection. We previously showed that macaques infected with SHIV during PrEP with FTC/TDF display reduced acute plasma viremias and limited virus diversity. We investigated the effect of PrEP on acute SHIV DNA dynamics and on the size of the persistent virus reservoir in lymphoid tissues.

**Design:**

Cell-associated SHIV DNA levels in PBMCs were measured in 8 macaques infected during PrEP with FTC/TDF or single-agent TAF and was compared to those seen in untreated infections (n = 10). PrEP breakthrough infections continued treatment with 1–2 weekly drug doses to model suboptimal drug exposure during undiagnosed HIV infection in humans. SHIV DNA was also measured in lymphoid tissues collected from FTC/TDF PrEP breakthroughs after 1 year of infection.

**Results:**

Compared to untreated controls, PrEP infections had reduced plasma RNA viremias both at peak and throughout weeks 1–12 (p<0.005). SHIV DNA levels were also reduced at peak and during the first 12 weeks of infection (p<0.043) but not throughout weeks 12–20. At 1 year, SHIV DNA reservoirs in lymphoid tissues were similar in size among macaques that received PrEP or placebo.

**Conclusions:**

Antiviral drug activity due to PrEP limits acute SHIV replication but has only a transient effect on cell-associated SHIV DNA levels. Our model suggests that suboptimal drug exposure in persons that are taking PrEP and become infected with HIV may not be sufficient to reduce the pool of HIV-infected cells, and that treatment intensification may be needed to sustain potential virological benefits from the PrEP regimen.

## Introduction

Daily pre-exposure prophylaxis (PrEP) with tenofovir disoproxil fumarate (TDF) in combination with emtricitabine (FTC) is an effective strategy to prevent HIV infection. In clinical trials, the level of HIV protection by PrEP was strongly correlated with drug adherence [1]. In the Partners PrEP trial among heterosexual men and women, plasma tenofovir (TFV) concentrations consistent with daily FTC/TDF dosing were associated with a protective effect of 91% [2]. A modelling study combining data from the iPrEX trial in men or transgender women who have sex with men and the STRAND study with directly observed dosing predicted HIV reductions of 76%, 96% and 99% if FTC/TDF were dosed at 2, 4, and 7 doses per week [3]. Very low adherence was one of the reasons why two other clinical trials among young African women (VOICE and FEM-PrEP) failed to show any efficacy of either daily TDF or FTC/TDF [4, 5]. Across trials, analyses of participants with detectable drug levels demonstrated higher efficacy than analyses that did not include objective adherence measurements.

Suboptimal PrEP adherence and HIV infection creates an opportunity for continued antiretroviral (ARV) drug activity during undiagnosed infection. Transient drug activity between HIV infection and diagnosis may potentially reduce virus replication. In macaques, infection with a chimeric simian HIV (SHIV) during concurrent PrEP failure with FTC/TDF resulted in blunted acute plasma viremias documented sequentially by weekly sampling following first viral RNA detection [6, 7]. In addition, a genetically more homogeneous virus populations was found in these macaques compared to untreated infections [8]. This virological outcome also resulted in a better preservation of immune responses [9]. Limited sampling in human PrEP studies may not allow the demonstration of blunted viremias in persons who fail PrEP, however, in a small analysis of seroconverters from the TDF2 human PrEP trial, HIV sequences from participants with measurable FTC and TFV in plasma at their seroconversion visit were found to be more homogeneous than those from participants who had no detectable FTC or TFV or who were not taking PrEP [10]. Although not definitive, these findings suggested that transient drug activity during PrEP breakthrough infection with either HIV or SHIV can decrease virus replication and slow viral evolution.

Emerging evidence shows that early ARV treatment has greater impact in limiting HIV or SIV reservoirs than later treatment but the long-term effect on virus control or total reservoir size is not fully clear especially when ARV treatment is stopped [11–14]. Here we used a well-established SHIV PrEP model with well-defined PrEP regimens and durations of ARV treatment to retrospectively investigate the impact of transient ARV exposure maintained during PrEP failure on the size of the cell-associated SHIV DNA reservoir. We first defined acute SHIV DNA dynamics in PBMCs from macaques that failed PrEP but continued receiving ARV treatment with 1–2 weekly doses of FTC/TDF combination or the single agent tenofovir alafenamide (TAF). We also investigated the long-term effects of PrEP on SHIV DNA pools by measuring the size of DNA reservoirs in lymphoid tissues late in the infection after ARV treatment was stopped.

## Material and Methods

### Ethics statement

All animal procedures were performed according to NIH guidelines and approved by the Institutional Animal Care and Use Committee (IACUC) of the Centers for Disease Control and Prevention (CDC). Macaques were housed at the CDC under the care of CDC veterinarians in accordance with the Guide for the Care and Use of Laboratory Animals 8th Ed. All procedures were performed under anesthesia (10 mg/kg ketamine or 2–6 mg/kg telazol; intramuscular), and all efforts were made to minimize suffering, improve housing conditions, and to provide enrichment opportunities. For housing, macaques were maintained in cages that met or exceeded the minimum size requirements as stipulated in the Guide for the Care and Use of Laboratory Animals [cage dimensions (inches): 30 (height) x 30 (width) x 30 (length)]. Steps were taken to reduce animal suffering, which included providing enrichment opportunities [e.g. cage features like swings/perches and objects for the macaques to manipulate], an assortment of food selections like fruits, vegetables or seeds, suitable feeding methods (foraging and task-oriented), and humane interactions with caregivers and research staff. All animals have access to clean, fresh water at all times. Commercial diets are specifically formulated to meet vitamin C requirements. Prior to the initiation of virus inoculations, compatible macaques are pair-housed. Once inoculations are initiated, the macaques are separated into single housing (while permitting eye-contact) with a cage divider to prevent the possibility of SHIV transmission between the macaques. If one macaque remained uninfected during the course of a study, the animal remained separated from other infected macaques, and was not be pair-housed with an infected macaque during the follow-up period. Euthanasia of SHIV infected macaques (n = 30 from studies in references [6, 7, 15, 16]) was accomplished in a humane manner (intravenous pentobarbital) by acceptable techniques as recommended by the American Veterinary Medical Association Guidelines on Euthanasia, 2013, and in accordance with CDC-Atlanta IACUC Policy on Euthanasia. The senior medical veterinarian and/or trained Comparative Medicine Branch staff verified successful euthanasia by the lack of a heart beat and respiration. Death was not used as a study endpoint.

### Study population

A first cohort of macaques comprised animals enrolled in SHIV transmission studies as PrEP-treated (n = 8) or controls (n = 10) [6, 7, 15]. These macaques were exposed rectally to SHIV_162P3_ once a week for up to 14 weeks until infection was confirmed by molecular and serologic testing [6, 7, 15]. The PrEP regimens consisted of two weekly doses of FTC/TDF (n = 6) or one weekly dose of TAF (n = 2). The doses of FTC/TDF were given i) 24h before and 24h after (-24/+24h) exposure, ii) 3 days before and 2h after (-3d/+2h) exposure, or iii) 7 days before and 2h after (-7d/+2h) exposure [6]. The macaques infected during TAF PrEP received a single dose of TAF 3 days before SHIV challenge (-3d) (Table 1) [15]. All 8 macaques with PrEP breakthrough infections continued receiving drug for an average of 20 (11–28) weeks after confirmed infection (Table 1).

**Table 1:** Treatment dosing schedule and duration of treatment in PrEP breakthrough infections.

	Treatment	Number of weekly drug doses (dosing schedule)	Duration of treatment after infection (months)
**33053**	FTC/TDF	2 (-24h/+24h)	7.0
**FCW**	FTC/TDF	2 (-24h/+24h)	6.5
**35838**	FTC/TDF	2 (-24h/+24h)	6.0
**DK40**	FTC/TDF	2 (-3d/+2h)	5.0
**34912**	FTC/TDF	2 (-7d/+2h)	2.5
**35032**	FTC/TDF	2 (-7d/+2h)	5.3
**DL2L**	TAF	1 (-3d)	2.8
**1784**	TAF	1 (-3d)	3.0

A second cohort of 17 animals was used to investigate the long-term effect of early PrEP treatment. We quantified cell-associated SHIV DNA in lymphoid tissues (mesenteric, axillary, and inguinal lymph nodes) collected at necropsy approximately 1 year after confirmed infection. Of these animals, 9 were infected during intermittent PrEP with FTC/TDF and 8 were untreated controls. Because of limited specimen availability, only a few animals from the first cohort were included in this second analysis. We thus included additional animals that also received FTC/TDF (1 or 2 doses every week) during the first months (median = 3.7) of infection [6, 16] (Table 3). Some of the macaques received a single dose of FTC/TDF or TAF 1–3 days prior to necropsy to measure drug penetration in rectal tissues [6, 15]. Lymph nodes were collected 11 months (FTC/TDF) and 9 months (untreated controls) after confirmed infection and were processed as previously described [15]. Briefly, the tissues were homogenized with a cell strainer (Fine mesh screens #20 and #10; company), washed several times with RPMI 1640, and then carefully layered over Ficoll (Lymphocyte Separation Medium (LSM, MP Biomedicals, Aurora, OH)) to separate lymph node mononuclear cells (LNMCs). LNMCs were subsequently washed with RPMI 1640, counted, and stored in liquid nitrogen until use.

### Quantitation of total SHIV DNA using a double-stranded primer (DSP) assay

Rhesus macaque PBMCs were obtained from blood collected in BD Vacutainer® CPT™ Cell Preparation Tubes (CPT) containing sodium heparin, resuspended in 200 ul of PBS, and stored at -80°C until use. Genomic DNA was extracted from 200 ul aliquots using the QIAamp DNA Mini and Blood Mini kit (Qiagen) and resuspended in 100 ul of elution buffer. Total SHIV DNA was quantified using a novel DSP assay coupled with co-amplification of RNase P gene as internal PBMC control [17]. Amplification of SIV LTR (52 bp) was done using primers pp119F (5’- Cy5/Quas670-GCCCTGGGAGGTTCTCTCCAGC-3’) and pp119R; (5’- GCAGGGAACACCCAGGCTCTAC-3’) with quencher probe pp119Q (5’- GAGAACCTCCCAGGGC-3’) with BHQ3 at the 3’ end. The final concentrations of primers and probe were 0.15uM and 0.37uM, respectively. Co-amplification of RNase P (53 bp) was done using primers cp1116 (5’-FAM-CGGTGTTTGCAGACTTGGACGTG-3’) and cp1118 (5’- GCGCAGAGCCTTCAGGTCAGA-3’) with quencher probe cp117 (5’- CCAAGTCTGCAAACACCG-3’) with BHQ2 at the 3’ end. Final primers and probe concentrations for RNase P were 0.07uM and 0.19uM, respectively. PCR amplification was done in an iQ5 Thermal Cycler (Bio-Rad) using the Quantifast Multiplex Kit (Qiagen, Valencia, CA), and included activation of DNA polymerase at 95°C for 90 seconds followed by 42 cycles of 94°C for 1 second and 63°C for 20 seconds.

SHIV_162P3_ DNA and cellular RNase P were quantified using standard curves prepared by serially diluting known copies of plasmid pVP1 and known amounts of macaque PBMCs, respectively. Plasmid pVP1 contains the 5′ portion of SIV_mac239_ from the 5′ long terminal repeat to a Sph I restriction site at nucleotide 6707, and was kindly provided by Dr. Cecilia Cheng-Mayer (Aaron Diamond AIDS Research Center). The concentration of pVP1 was determined using a Nano Drop ND-1000 Spectrophotometer. Control PBMCs were prepared from uninfected rhesus macaques and counted using the Guava Personal Cell Analysis (PCA) (Millipore, Billerica, MA) system. S1 Fig shows that the DSP assay was able to consistently detect 10 copies of pVP1 plasmid (10/10 replicates positive). Assay reproducibility was investigated by measuring variability in Ct values obtained in 16 separate dilution series prepared from 100,000 to 10 copies of pVP1 plasmid and from 300,000 to 30 rhesus PBMCs. The percentage coefficient of variation (CV) was <4% for all different SIV DNA or PBMC inputs, demonstrating the robustness of the DSP assay in quantifying SHIV DNA and cellular RNase P with a wide dynamic range (S1 Fig).

### Quantification of SHIV RNA in plasma by one-step RT-PCR

SHIV_162P3_ RNA was quantified using a modification of an existing two-step RT-PCR assay [18] that includes the use of a single RT-PCR reaction. Prior to RNA extraction, plasma samples (1 ml) were centrifuged at 43,000xg for 30 min to concentrate virus particles. RNA was extracted from 140 ul virus pellets using the QIAamp Viral RNA kit (Qiagen) and eluted in 100 ul of elution buffer. To control for the efficiency of extraction, a known amount of virus particles (3×10^5^) from an HIV-1_CM240_ virus stock (NIH Reference Reagent Program) was added to each sample prior to RNA extraction.

Reverse transcription and PCR amplification of SHIV_162P3_ (gag) and HIV-1_CM240_ (env) sequences was done using primers specific for SIV_mac239_ and HIV-1_CM240_, respectively. SIV gag sequences were amplified using primers SIVp15f1 5’ GCC AAC AGG CTC AGA AAA TTT AA 3’ and SIVp15r (5’ TCC TCA GTG TGT TTC ACT TTC TCT TC 3’) with internal probe P12P HEX (5’HEX AGC CTT TAT AAT(BHQ1) ACT GTC TGC GTC ATC TGG TGC BHQ1 3’). Control HIV1_CM240_ was amplified using primers envE2f (5’ -GGA CAG GGC CAT GTA AAA ATG T -3’) and EnvE2r (5’ -TCT TCT GCT AGA CTG CCA TTT AAC AG -3’) with internal probe envEP (5’ FAM CAC ACA TGG AAT(BHQ1) TAA GCC AGT GRT ATC MAC TCA BHQ1 3’). RT-PCR was done using the Superscript III one-step qRT-PCR kit (Invitrogen). The final concentrations of SIV_mac239_ primers and probe in the reaction were 200 nM. Likewise, final concentrations of HIV-1_CM240_ primers and probe were 25nM and 100nM, respectively. Cycling conditions were 10 minutes at 25°C, 60 minutes at 48°C, and 45 cycles of 95°C for 15 seconds and 60°C for 1 minute. The sensitivity of the RT-PCR assay is 50 RNA copies/ml [19, 20].

### Statistical analysis

Peak RNA and DNA levels, area under the curve (AUC) values for RNA and DNA during 5 or 20 weeks, and tissue DNA levels in PrEP breakthrough and placebo controls were compared using a two-tailed Wilcoxon rank-sum test. All analyses were done in GraphPad software (version 5.04). The relationship between plasma RNA and cell-associated DNA levels was determined using the Pearson correlation coefficient.

## Results

### Acute plasma RNA and cell-associated SHIV DNA dynamics in macaques infected with SHIV_162p3_

**Fig 1:**
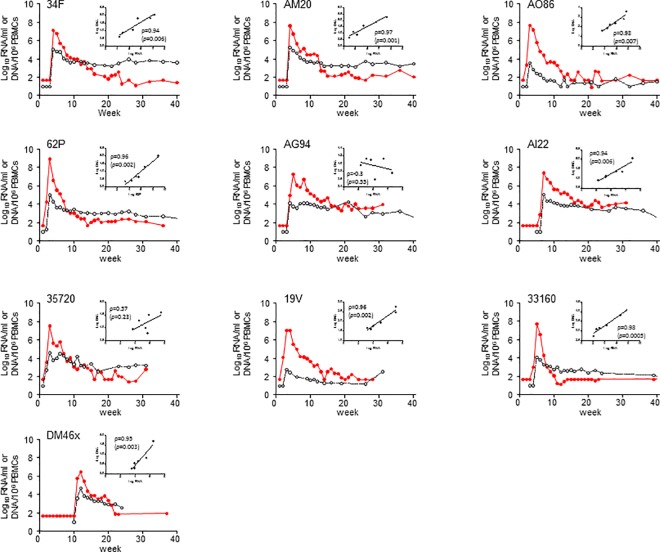
Acute plasma RNA and cell-associated SHIV DNA levels in macaques infected with SHIV_162P3_. Time 0 denotes peak plasma viremia. SHIV RNA levels in plasma are shown in red. Cell-associated SHIV DNA levels in PBMCs are shown in black. Small panels indicate the correlation between plasma RNA and DNA levels seen between peak viremia and week 5. Pearson correlation coefficients are also shown.

We first defined acute plasma RNA and cell-associated SHIV DNA dynamics in rhesus macaques (n = 10) infected rectally with SHIV_162P3_ without any PrEP intervention (Fig 1). The median (range) peak SHIV RNA viremia was 7.5 (6.5–8.9) log_10_ RNA copies/ml with RNA levels reaching a set-point at about 12 weeks of infection. Likewise, SHIV DNA levels peaked at 4.6 (2.8–5.3) log_10_ DNA copies/10^6^ PBMCs and reached a set-point generally within 5 weeks of infection. During these 5 weeks, DNA and RNA levels were highly correlated (Fig 1); Pearson correlation coefficients (ρ) were indicative of a very strong correlation (between 0.93 and 0.98) in 8 macaques, and of a strong correlation (ρ = 0.57) in 1 animal. Thus, infection of macaques with SHIV_162p3_ is characterized by a rapid control of plasma viremia within 12 weeks of infection and the establishment of a DNA reservoir of about 10^2^ to 10^4^ SHIV DNA copies per 10^6^ PBMCs.

### Effect of PrEP on acute SHIV DNA dynamics

To investigate if PrEP can reduce the levels of cell-associated SHIV DNA in PBMCs, we compared acute SHIV DNA dynamics between 8 PrEP breakthrough infections and the 10 untreated controls. These macaques were infected after a median of 5 (range = 2–14) rectal challenges which was not different from the median of 3 (range = 1–10) rectal challenges required to infect the 10 control animals (p = 0.099). Six of the 8 breakthrough infections occurred during intermittent PrEP with FTC/TDF (2 FTC/TDF doses per week) and two infections happened during intermittent PrEP with TAF (1 dose of TAF per week given 3 days prior to virus challenges). The 8 PrEP breakthrough infections continued receiving intermittent antiretroviral treatment after confirmed infection for an average of 4.7 months (Table 1). Treatment was originally maintained to investigate risks of drug resistance emergence although none of these animals developed the K65R or M184V mutation [6, 7, 15].

**Fig 2:**
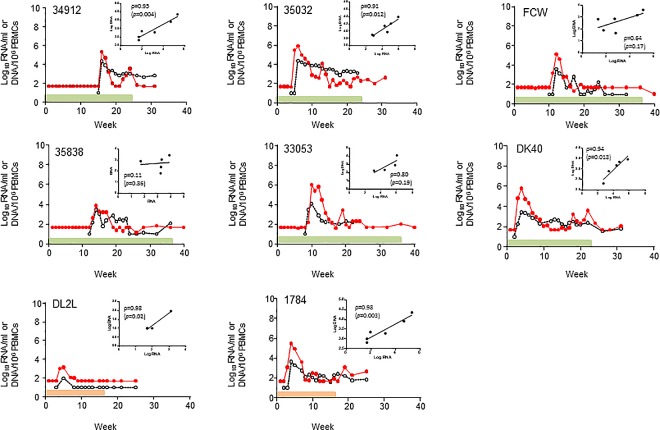
Acute plasma RNA and cell-associated SHIV DNA levels in macaques infected with SHIV_162P3_ while receiving PrEP. Time 0 denotes peak plasma viremia. SHIV RNA levels in plasma are shown in red. Cell-associated SHIV DNA levels in PBMCs are shown in black. The shaded areas denote the length of treatment (green = FTC/TDF; orange = TAF). Small panels indicate the correlation between plasma RNA and DNA levels seen between peak viremia and week 5. Pearson correlation coefficients are also shown.

Fig 2 shows plasma RNA and cell-associated SHIV DNA levels in the 8 PrEP breakthroughs infections and also highlights the length of treatment for each individual macaque. As seen in untreated SHIV_162p3_ infections, RNA and DNA levels were highly correlated during the first 5 weeks of infection with the only exception of macaques 35838 and 33053; Pearson correlation coefficients were very strong in 5 macaques (34912, 35032, DK40, DL2L, and 1784) and strong in 1 animal (FCW).

**Fig 3:**
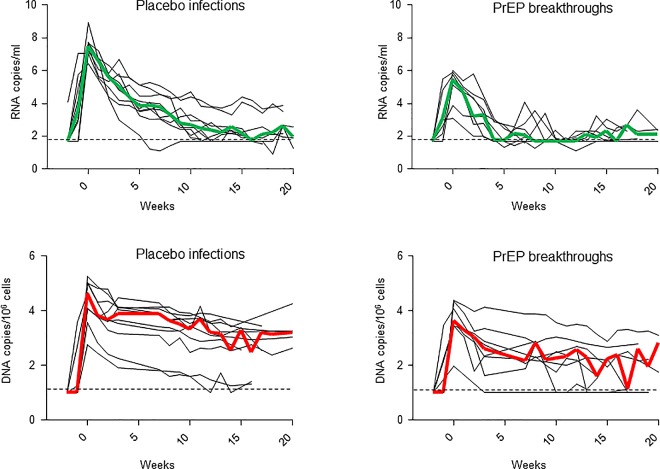
Acute viral RNA and cell-associated DNA levels in macaques infected with SHIV_162P3_ while receiving PrEP of placebo. The two top panels show plasma RNA levels in placebo and PrEP breakthrough infections, and the two panels at the bottom show cell-associated DNA levels in PBMCs. Time 0 denotes peak viremia. The green and red lines indicate median RNA or DNA levels, respectively. Horizontal dotted line denotes the limit of detection of the SHIV RNA or DNA assays.

Table 2 shows the median (range) values for SHIV RNA and DNA observed at peak, as well as the overall levels of RNA and DNA seen throughout weeks 0–12 or 12–20 of infection expressed as area under the curve (AUC) values. These intervals were selected to cover the acute phase of infection with SHIV_162p3_ as well as the more chronic phase when viral RNA set-points are reached (generally about 12 weeks; Fig 3 and [7]). Peak SHIV DNA levels in PBMCs in PrEP breakthrough infections (3.6 log_10_ DNA copies/10^6^ cells; range = 2.0–4.4) were significantly lower than in untreated controls (4.6 log_10_; range = 2.8–5.3) (*p* = 0.043). DNA levels during the first 12 weeks of infection were also lower in the animals that received PrEP (AUC_0-12wk_ of 34.3 compared to 49.3 in controls; *p* = 0.043). (Table 2). However, DNA levels throughout weeks 12–20 were not significantly different from those seen in the control group (AUC_12-20wk_ values of 11.7 and 19.6; *p* = 0.274) (Table 2). These results demonstrate a transient effect of PrEP on the SHIV DNA reservoir that is not sustained during intermittent drug treatment and wanes over time. As noted in previous studies [6, 8, 15], peak RNA levels and early acute RNA viremias (AUC_0-12wk_) were also significantly lower in PrEP breakthrough infections compared to untreated infections (*p* < 0.0001 and *p* = 0.0003, respectively, Table 2). The difference in RNA levels was not evident throughout weeks 12–20, likely reflecting the low RNA set points that are generally achieved in rhesus macaques infected mucosally with SHIV_162P3_ (Table 2 and Fig 3).

**Table 2:** SHIV_162P3_ plasma RNA and cell-associated DNA in PrEP breakthroughs and placebo infections.

	PrEP breakthrough infections (n = 8)	Placebo Infections (n = 10)	
**SHIV DNA in PBMCs**			
Peak	3.6 (2.0–4.4)	4.6 (2.8–5.3)	p = 0.043
AUC_0-12wks_	34.3 (15.4–50.7)	49.3 (25.9–54.3)	p = 0.043
AUC_12-20wks_	11.7 (4.5–25.9)	19.6 (5.2–30.3)	p = 0.274
**SHIV Plasma RNA**			
Peak	5.4 (3.1–6.0)	7.5 (6.5–8.9)	p<0.0001
AUC_0-12wks_	37.7 (27.9–48.2)	57.9 (39.8–71.1)	p = 0.0003
AUC_12-20wks_	12.7 (5.1–21.3)	17.2 (8.4–27.6)	p = 0.159

We also evaluated if the overall SHIV DNA levels were related to the number of challenges needed to infect a macaque. No significant correlation was found between AUC_0-12wk_ or AUC_12-20wk_ values and cumulative number of virus challenges (Pearson correlation coefficients of -0.128 (*p* = 0.61) and -0.384 (*p* = 0.115), respectively).

### Long-term effect of PrEP on SHIV DNA levels in lymphoid tissues

We next investigated the effect of PrEP on the size of the virus reservoir in lymphoid tissues in a late-stage of infection. We quantified cell-associated SHIV DNA in mesenteric, axillary, and inguinal lymph nodes collected from 9 PrEP breakthrough and 8 placebo infections at necropsy. The specific PrEP interventions and dosing regimens, the length of treatment and time between SHIV infection and necropsy, and the levels of SHIV DNA for each lymph node location are shown in Table 3. Because of limited specimen availability, only a few animals from the first cohort were included in this second analysis. However, peak RNA viremias and AUC_0-12wk_ values in these additional PrEP breakthrough infections were also reduced compared to untreated controls (*p* = 0.015 for each comparison).

**Table 3:** SHIV_162P3_ DNA levels in lymphoid tissues from PrEP breakthrough and placebo infections.

				Log_10_ SHIV DNA copies/10^6^ cells		
	Number of weekly FTC/TDF doses (dosing schedule)	Length of treatment (months)	Months of infection prior to necropsy	Axillary LN	Inguinal LN	Mesenteric LN
**Untreated**						
G47	-	-	6	3.1	2.8	2.9
A2E027	-	-	6	3.4	3.3	3.2
A4E037	-	-	6	4.4	n.a.	4.4
79R	-	-	17	3.1	3.0	2.9
19V	-	-	9	n.a.	0.7	0.7
AG65	-	-	17	2.6	2.4	2.7
34F	-	-	18	3.5	3.2	3.6
RKZ7	-	-	9	3.2	2.9	2.9
*Median*			*9*	*3*.*2*	*2*.*9*	*2*.*9*
**FTC/TDF**						
1800	2 (-2h/+22h)	4.3	15	2.9	n.a.	3.0
DM91	2 (-2h/+22h)	4.3	16	3.1	3.0	n.a.
DL6V	2 (-22h/+2h)	3.8	16	0.7	2.6	2.4
FCW	2 (-24h/+24h)	6.5	10	0.7	1.8	1.6
DK40	2 (-3d/+2h)	5.0	11	2.6	2.3	2.5
34912	2 (-7d/+2h)	2.5	12	2.7	2.5	2.3
DL2T	1 (-3d)	3.8	8	1.6	0.7	1.6
35489	1 (-3d)	3.5	8	3.2	3.0	3.9
DK2R	1 (-3d)	3.5	6	n.a.	n.a.	3.7
*Median*		*3*.*7*	*11*	*2*.*6*	*2*.*5*	*2*.*5*
			*p* = 0.91	*p* = 0.02	*p* = 0.27	*p* = 0.44

n.a., not available

**Fig 4:**
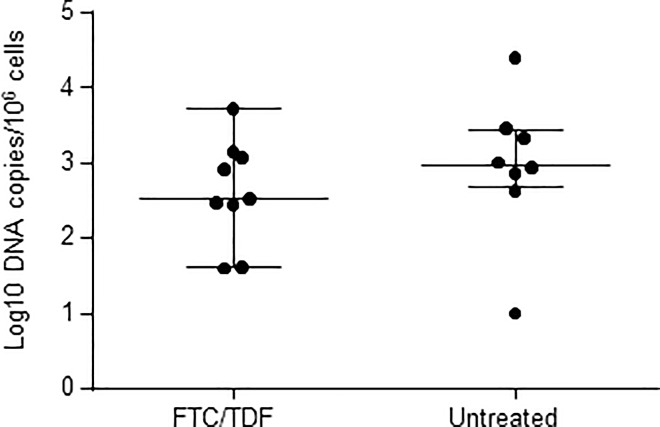
SHIV_162P3_ DNA levels in lymphoid tissues collected from PrEP breakthrough infections and untreated controls. Each individual point corresponds to the median SHIV DNA value observed in mesenteric, axillary, and inguinal lymph nodes from one macaque. Overall SHIV DNA levels in FTC/TDF PrEP breakthrough infections were not significantly different from those seen in placebo infections (*p* = 0.34). Horizontal lines are median values observed in each group. Vertical lines are ranges.

Overall, SHIV DNA reservoirs in lymphoid tissues were similar in size among macaques that received PrEP with FTC/TDF (median = 2.65 log_10_ SHIV DNA copies/10^6^ cells; range = 1.6–3.7) or placebo (median = 2.98; range = undetectable-4.4) (*p* = 0.34) (Fig 4). When individual LN were analyzed, we found lower levels of SHIV DNA in axillary lymph nodes collected from PrEP breakthrough infections (2.6 vs 3.2 DNA copies/10^6^ cells in controls, *p* = 0.02). Levels of SHIV DNA in inguinal and mesenteric lymph nodes were comparable among the 2 groups of macaques (Table 3).

## Discussion

We investigated in macaques if transient drug activity during PrEP breakthrough infection has the potential to alter the pool of SHIV-infected cells. Our analysis included macaques that received non-daily PrEP regimens with either FTC/TDF combination or TAF, and that continued receiving treatment with 1–2 weekly pills during several months. This scenario can easily mimic newly acquired HIV-1 in persons receiving PrEP due to inconsistent adherence and who continue on the PrEP regimen until diagnosis. We found that PrEP significantly reduced peak SHIV DNA levels in PBMCs, although the overall effect on the cell-associated DNA reservoir was only transient and was lost during the first year of infection. Our findings differ from studies of acute treatment with fully suppressive regimens which have generally observed substantial reductions in cell-associated HIV or SIV DNA in PBMCs and lymphoid tissues [11–14]. We surmise that antiretroviral drug activity when PrEP fails may not provide adequate drug selective pressure to effectively reduce the pool of HIV-1-infected cells and result in post-treatment virus control.

Our analysis was retrospective and included a heterogeneous group of macaques that received FTC/TDF or TAF at different times relative to virus challenges. However, most of the FTC/TDF failures received 2 weekly drug doses which is sufficient for accumulating TFV-DP in PBMCs at levels above the prophylactic EC_95_ in macaques [21]. The dose of TAF given to two of the animals was also sufficient to sustain high (>1000 fmols/10^6^ cells) TFV-DP concentrations in PBMCs for up to 7 days [15]. Thus, although from different PrEP modalities, our approach effectively models transient antiviral drug activity that originates from failed PrEP regimens during acute infection.

In contrast to persons who fail PrEP because of low medication adherence and drug levels, our study included animals who represented rare PrEP failures that had drug concentrations in PBMCs that were indistinguishable from protected animals [6, 7]. For instance, macaque DK40 was the only animal infected during a two-dose FTC/TDF PrEP regimen that protected 5 other animals for a cumulative of 70 SHIV challenges [6]. Likewise, macaques 34912 and 35032 became infected while receiving an FTC/TDF regimen that decreased the risk of SHIV infection by a factor of 9 [6]. Thus, the reductions in peak RNA and DNA levels seen in our animals may not necessarily occur in HIV infections that are acquired due to low medication adherence and substantially lower drug levels at the time of HIV acquisition. At later time points after infection, continuous intermittent treatment with 1 or 2 weekly doses of FTC/TDF or TAF effectively models suboptimal drug adherence during undiagnosed infection, and was the likely reason for the partial reductions in SHIV plasma viremia. Although viremias were generally suppressed during the period of treatment, the replenishment of the cell-associated SHIV DNA pool demonstrated expanded virus replication and seeding of virus reservoirs and, thus, absence of post-treatment virus control despite the early reduction in virus replication.

Although not statistically significant, we found that the number of SHIV challenges required to infect untreated macaques was smaller than those required to infect the animals that failed PrEP, which raises questions about the effect of previous SHIV exposures on systemic control of the virus post-infection. In a large retrospective analysis of 40 rhesus macaques exposed repeatedly to SHIV_162p3_, we found no correlation between cumulative exposures to SHIV and peak plasma RNA levels or AUC values [22]. In our study we also found no correlation between cumulative SHIV exposures and SHIV DNA levels during the acute or the plateau phase, suggesting that previous virus exposures have little or no impact on cell-associated DNA levels in PBMCs.

The present study has several limitations. First, we did not address potential effects of PrEP on the fraction of DNA genomes that are replication-competent. Reduced virus replication and evolution due to PrEP might also result in more genetically homogeneous virus reservoirs or favor preferential seeding of sequences with replication advantages in the presence of FTC or TFV [8]. Second, we measured total cell-associated DNA which includes unintegrated DNA that may degrade more rapidly and contribute little to viral production. Also, we did not assess reservoir distribution in cell subsets. In humans, early HAART is associated with lower infection rates of cell subsets with long life span as naïve and central memory T cells and a better preservation of immune function [11, 23, 24]. Finally, although SHIV_162p3_ is easily transmissible at low doses and is thus well-suited for transmissibility studies, infections with this isolate generally result in low set-point viremias compared to more pathogenic SIV isolates. Also, the infection course is generally non-pathogenic [25, 26] which limits the ability to define long-term effects of PrEP after treatment discontinuation.

In summary, we show that transient antiviral drug activity due to PrEP limits acute SHIV replication but only has a transient effect on the size of the DNA reservoirs. These observations suggest that suboptimal drug exposure in persons that are taking PrEP and become infected with HIV may not be sufficient to durably reduce the pool of HIV-1-infected cells and, therefore, not likely to predict good virus control post PrEP interruption.

## Supporting Information

S1 Fig Sensitivity and inter-assay variability of the DSP SHIV DNA assay. A) Serial dilutions of SIV vP1 DNA plasmid were prepared in PBS and tested 10 times in the same run. The assay consistently detected 10 copies of vp1. (B) Variability in Ct values observed in 16 separate runs at different inputs of SIVmac239 vp1 plasmid and rhesus PBMCs. In all instances the % coefficient of variation (CV) was <4%.
(TIF)
