# Supplementary figures and images for: Antiretroviral Drug Activity in Macaques Infected during Pre-Exposure Prophylaxis Has a Transient Effect on Cell-Associated SHIV DNA Reservoirs
Source: PLoS One. 2016 Nov 2;11(11):e0164821. doi: 10.1371/journal.pone.0164821 (PMC5091888; doi:10.1371/journal.pone.0164821)

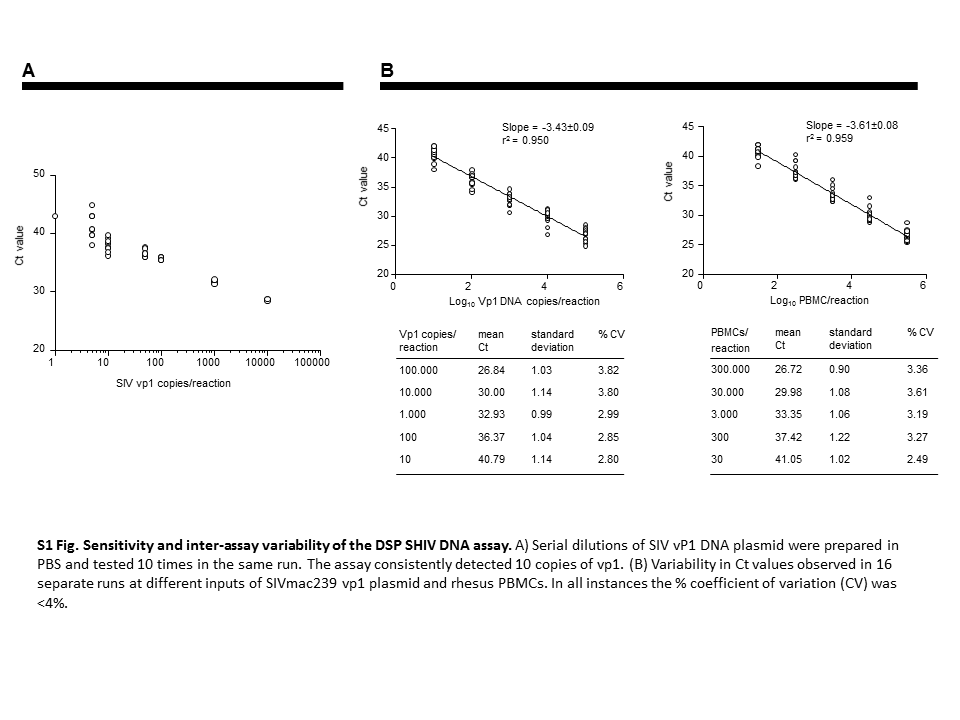

Supplement: S1 Fig — A) Serial dilutions of SIV vP1 DNA plasmid were prepared in PBS and tested 10 times in the same run. The assay consistently detected 10 copies of vp1. (B) Variability in Ct values observed in 16 separate runs at different inputs of SIVmac239 vp1 plasmid and rhesus PBMCs. In all instances the % coefficient of variation (CV) was <4%. (TIF) [file pone.0164821.s001.tif]
